# Drug-drug relationship based on target information: application to drug target identification

**DOI:** 10.1186/1752-0509-5-S2-S12

**Published:** 2011-12-14

**Authors:** Keunwan Park, Dongsup Kim

**Affiliations:** 1 Department of Bio and Brain Engineering, KAIST, 373-1, Guseong-dong, Yuseong-gu, Daejeon, 305-701, Republic of Korea

## Abstract

**Background:**

Drugs that bind to common targets likely exert similar activities. In this target-centric view, the inclusion of richer target information may better represent the relationships between drugs and their activities. Under this assumption, we expanded the “common binding rule” assumption of QSAR to create a new drug-drug relationship score (DRS).

**Method:**

Our method uses various chemical features to encode drug target information into the drug-drug relationship information. Specifically, drug pairs were transformed into numerical vectors containing the basal drug properties and their differences. After that, machine learning techniques such as data cleaning, dimension reduction, and ensemble classifier were used to prioritize drug pairs bound to a common target. In other words, the estimation of the drug-drug relationship is restated as a large-scale classification problem, which provides the framework for using state-of-the-art machine learning techniques with thousands of chemical features for newly defining drug-drug relationships.

**Conclusions:**

Various aspects of the presented score were examined to determine its reliability and usefulness: the abundance of common domains for the predicted drug pairs, c.a. 80% coverage for known targets, successful identifications of unknown targets, and a meaningful correlation with another cutting-edge method for analyzing drug similarities. The most significant strength of our method is that the DRS can be used to describe phenotypic similarities, such as pharmacological effects.

## Introduction

Recently, many studies have examined the quantitative structure-activity relationship (QSAR) between drugs, as researchers seek to characterize chemical compounds in terms of their activities. Thus far, the studies have adopted a mathematical procedure which transforms chemical properties into numeric features, the so-called “molecular descriptor.” Until now, many thousands of descriptors have been devised and have proven to be useful for predicting a variety of drug activities, such as drug-likeness [1], pharmacokinetic parameters [2], acute toxicity [3], multi-modal binding propensity [4], and many other physicochemical properties [5] (e.g. log P). Furthermore, descriptors have also been used to infer the drug-drug relationship, which expands the applicability to virtual screening [6,7], chemical library construction [8], drug clustering [9] and classification [10-12].

The wide availability of chemical information (descriptors) is based on an implicit assumption that drugs that bind to the same target likely exert similar activities. In line with this thinking, the theory of “neighborhood behavior” [13] has long asserted that structurally similar drugs likely bind to a common therapeutic target. Therefore, it can be said that drug target information is the most direct evidence for inferring a drug’s activity. In this target-centric view, the inclusion of richer target information may better represent the relationships between drugs and their activities. However, drug-drug relationships have typically been calculated using chemical structural information [14-16]. That is, a chemical structure is converted into numerical features representing various chemical properties [17], and the structural features are then used to define the drug-drug relationship by determining which features are the same and which are different. However, the weak point of this method is that it cannot consider many structurally unrelated drugs bound to a common target [18,19].

In this study, we present a new drug-drug relationship score (DRS) which aims to encode both the drug target information and the global structural similarity. The “common binding rule” assumption of QSAR studies was used and expanded to posit the existence of common rules governing drug-target interaction which could be learned from large-scale drug-target interaction data.

Specifically, more than 2,000 descriptors were used to transform drug pairs into numerical vectors. The estimation of drug-drug relationships was thus restated in a classification framework that prioritizes drug pairs with a common target. This procedure was based on the assumption that drugs sharing a target are much more similar than drugs that are only alike in terms of structure. To improve the reliability of the score, data cleaning, iterative under-sampling, and the ensemble approach were combined with a Random Forest classifier.

The classification performance was validated using both an internal and external test set. In addition, the reliability and usefulness of the DRS were examined in terms of the abundance of common domains for the predicted drug pairs, c.a. 80% coverage for known targets, successful examples for unknown target identifications, and meaningful correlation with another cutting-edge technique. Significantly, the DRS showed better performance for describing similarity in pharmacological effects [8], perhaps due to the encoded target information.

## Results and discussion

### Generating drug-drug relationship score

**Figure 1:**
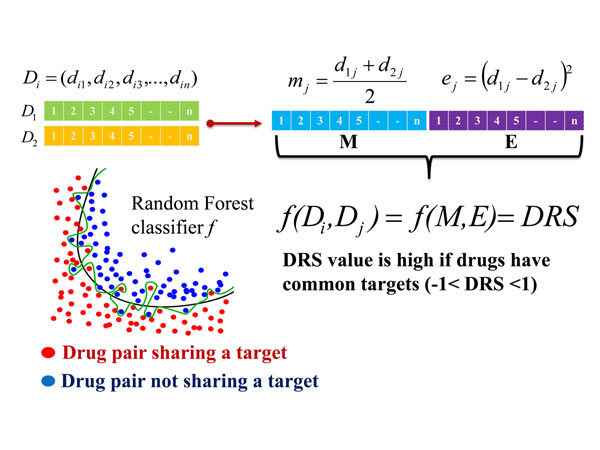
Construction of drug pair vector and the classification model using Random Forest are shown. For example, two drugs, D1 and D2, are represented by *n* principal components, and the resulting M (basal chemical properties) and E (chemical property differences) vectors are used to represent the drug pairs. The classification model classifies the positive drug pairs that share a target (red) from the negative drug pairs that do not share a target (blue).

To derive the DRS, a drug pair vector was constructed by averaging and subtracting paired drug features in descriptor space (Figure 1). All drug pairs were classified into two groups: positive drug pairs (which shared at least one common target) and negative drug pairs (which did not share any targets). After that, machine learning techniques were adopted to prioritize drug pairs bound to a common target (see the Methods section for the detailed procedures). Conceptually, this procedure implemented the assumption that drugs with common targets might have more similar actions than structurally similar drugs.

**Figure 2:**
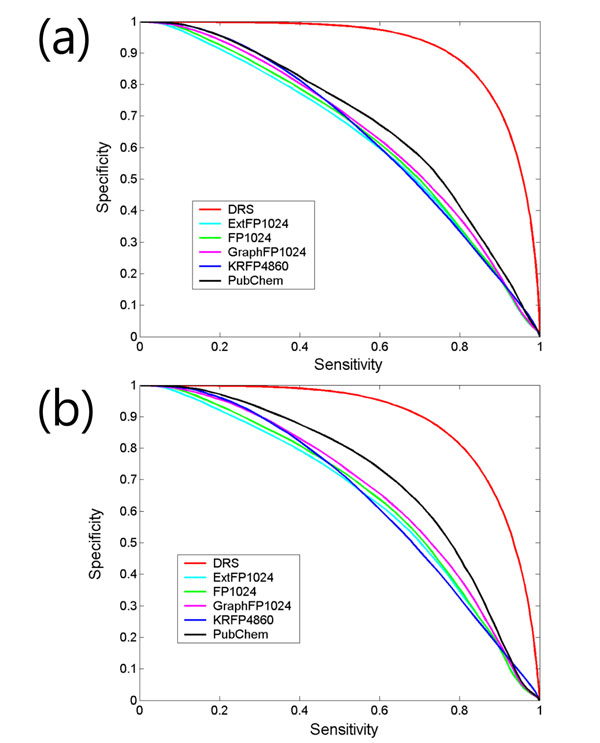
Specificity and sensitivity plot of (a) internal cross-validation using OOB samples and (b) external validation using an independent test set generated from 50 drugs excluded at the training step. The other drug similarity measures are compared with the drug-drug relationship score (DRS).

To estimate the classification proficiency, we performed internal cross-validation, using out-of-bag (OOB) samples, and external validation, using an independent test set. As a baseline method, 2D structural similarity measures based on the different fingerprints of the drugs were calculated and compared with the DRS. That is, the drug pairs were sorted by the Tanimoto coefficient and checked to see if they shared the same target. The performance is represented by the sensitivity-specificity plot in Figure 2. The results of internal cross-validation showed that the DRS outperformed the 2D similarity measures in retrieving common-target drugs (Figure 2a). When the score threshold was set to zero, the sensitivity and the specificity reached about 0.8 and 0.8, respectively. In addition, the results of external validation also showed a similar trend, even though the performance was a little bit lower than the internal cross-validation (Figure 2b).

These results suggest that the DRS contains more useful target information than traditional similarity measures, and the classification model seems to be unbiased by the huge amounts of negative data. In addition, true positives (correctly predicted drug pairs) covered many structurally-unrelated drug pairs (Additional file 1), implying that the DRS could capture the important spatial features of structurally-unrelated drug-pairs. On the other hand, the performances of the five structural similarity measures were virtually identical, although PubChem fingerprint showed the best performance.

### Predicted drug pairs seem to be promising: high domain-matching ratio

In the classification framework, drug pairs that do not share any known common targets were considered as negative data. However, it is possible that the drugs’ shared common targets might be unknown because of insufficient knowledge about drug-target interaction. Therefore, using the DRS to mine unknown drug-drug relationships could be very interesting work. Indeed, new similarities between drugs were used to reposition the marketed drugs by revealing unknown drug-drug relationship [20,21]. From this view point, drug pairs predicted as positives might have a better chance of sharing a common target than negative drug pairs.

**Figure 3:**
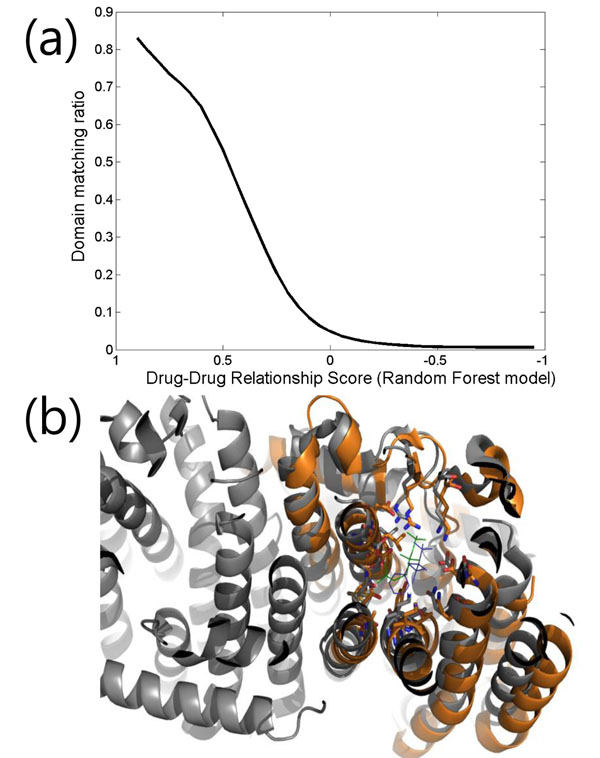
(a) PFAM domain matching ratio for the negative drug pairs is shown according to the drug-drug relationship score. (b) Example target structures of DB02270 (blue stick) and DB00884 (green stick) are shown as gray (1RQI) and orange (1YV5), respectively. Their RMSD value is 2.56Å, probably due to the common polyprenyl synthetase domain.

To estimate the hypothesis, the PFAM domains [22] of the targets of the negative drugs were investigated to see if the drug pairs had a target of the same domain (Figure 3a). It was assumed that drug targets of the same domains likely bind to the same drug because of their structural and sequential homology. For example, the structural similarity between DB02270 and DB00884 was very low (Tanimoto coefficient based on PubChem fingerprint: 0.15) in spite of a high DRS (0.77, when the range was adjusted from 0 to 1 as the structural similarity). The maximum target identity between possible target pairs was also relatively low (sequence identity: 23%). However, the overall target structures, especially ligand binding pockets, were very similar (Cα RMSD 2.56Å for PDB id 1YV5 and 1RQI) because they shared the same PFAM domain: polyprenyl synthetase (PF00348). Indeed, the binding modes of the drugs appeared very similar to one another (Figure 3b). In addition, many drug pairs with potential similar binding pockets could be discovered by the domain matching information.

Specifically, the proportion of negative drug pairs that shared common PFAM domains was investigated according to the DRS. Note that negative drug pairs are those without any common targets. The results showed that a higher DRS represented a higher domain-matching ratio. For example, more than 50% of drug pairs had common target domains when the DRS was set to 0.5, which was significantly higher than the random (less than 1%). Accordingly, the result of the domain matching ratio suggests that DRS might be useful for finding unknown drug-drug relationships.

### New target identification by drug-drug relationship score

The newly predicted positive drug pairs (i.e. false positives in terms of classification) were used to identify potential targets. The target identification scheme based on the maximum DRS transferred the information on drug-drug relationships to the drug targets (See Methods). This scheme was successful for about 80% of the known drug targets (Additional file 2). To estimate the target-finding capability for unknowns, the recently discovered drug-target interactions by Keiser et al. [20] were used as a test set. Note that drugs whose discovered targets were not annotated in the DrugBank database were used in this study [23]. This process was similar to finding new targets of known drugs. The tested drugs were DMT (DB01488), Motilium (DB01184), Xenazine (DB04844), Prantal (DB00729), Paxil (DB00715), Prozac (DB00472), and Rescriptor (DB00705), and their known targets are listed in Table 1, along with their DRS values and ranks. In addition, the target scores from the false positive drug pairs (those with a high DRS value but no common target) were separated from those of the known positive drug pairs (which shared a common target). Thus, this separation (Table 1) was designed to determine whether the new target predictions were meaningful.

**Table 1:** Drug target prediction examples by the DRS

Drug Name	Known Targets	Score (frequency for score ties) from the positive drug-pairs	Rank	Score (frequency for score ties) from the newly predicted drug-pairs (not sharing a target)	Rank
DMT (DB01488)	**5-HT7** **5-HT1B** **5-HT5A** 5-HT6 5-HT1D 5-HT1A σ1 5-HT2A 5-HT2C 5-HT2B	0.858(10) 0.858(14) NA 0.858(6) 0.858(16) 0.910 0.772 0.942(63) 0.942(32) 0.882	40 39 NA 42 38 17 52 1 3 32	0.426 0.778(4) NA NA 0.778(4) 0.529(2) NA NA 0.376 0.778(2)	75 3 NA NA 3 45 NA NA 99 5

	K+	0.939 [Potassium H2]	16	0.655 [Potassium D2,D3,KQT2,A1]	28
	hERG	NA	NA	NA	NA
	D2	0.961(63)	1	NA	NA
Motilium (DB01184)	D3	0.917	22	NA	NA
	**α1**	α1A 0.961(26)	4	α1B 0.700(1)	18
		α1B 0.961(20)	7	α1A 0.694	20
	5(HT2A)	0.961(41)	2	0.752	10

	VMAT2	0.878	1	NA	NA
Xenazine (DB04844)	**α2**	α2A 0.807	14	α2A 0.706	4
		α2B 0.758	15	α2B 0.650	37

Prantal (DB00729)	**δ** M3	0.975 0.986	21 1	0.830 NA	49 NA

	M1	0.954(71)	1	NA	NA
	M2	0.954(58)	2	NA	NA
	M3	0.954(53)	3	NA	NA
	**β1-**	0.918 [β1 adrenergic receptor]	31	0.635 [β1 adrenergic receptor]	24
Paxil (DB00715)	DAT	0.951(24)	10	0.775	4
	NET	0.951(45)	6	NA	NA
	5-HTT	0.951(42)	7	NA	NA
	α1	α1A 0.951(41)	8	α1A 0.687	15
		α1B 0.939	12	α1B 0.545	60

Prozac (DB00472)	5HTT NET **β1-** CA H1 5-HT2C 5-HT2A M1	0.992(42) 0.992(32) 0.988 [β1 adrenergic receptor] NA 0.992(25) 0.991(35) 0.992(63) 0.992(23)	2 4 18 NA 5 14 1 6	NA 0.861 0.699 [β1 adrenergic receptor] CA2, 0.280 0.776 NA NA 0.735(8)	NA 3 106 357 86 NA NA 89

Rescriptor (DB00705)	**H4** HIVRT *Gag-Pol	NA NA 0.847	NA NA 1	0.259 NA NA	47 NA NA

	PTGS2	0.923	1	NA	NA
	PDPK1	0.746	21	NA	NA
	CA2	NA	NA	0.824	1
	Kv	0.505 [Kv subfamily C member 4]	177	0.451 [Kv subfamily KQT member 1]	47
				0.451 [Kv subfamily E member 1]	48

**Bold**: new prediction by Keiser, **under bar**: known interaction in DrugBank, *: annotated in DrugBank but not in the study by Keiser, **Abbreviations**: 5HT, 5-hydroxytryptamine; 5-HTT, serotonin transporter; K+, potassium channel; hERG, human Ehter-a-go-go related gene channel; D1-4, Dopamine 1-4; α1-2, α adrenergics; VMAT2, vesicular monoamine transporter 2; δ, δ-opioid receptor, M1-3, muscarinics; β1-, adrenertic agonist; DAT, dopamine transporter; NET, noradrenaline transporter; CA, carbonic anhydrase, H1-4, histaminergics; HIVRT, HIV-1 reverse transcriptase; Gag-Pol, Gag-Pol polyprotein; PTGS2, Prostaglandin G/H synthase 2; PDPK1, 3-phosphoinositide-dependent protein kinase 1; Kv, voltage-gated potassium channels

For most drugs, the target prediction scheme employing the DRS worked well, even for the new targets discovered by Keiser. For example, alpha-1 type adrenergic, the target of Motilium, could be found in the fourth rank (with a score that was tied with the first rank). In addition, other targets such as potassium channel (K+) and serotonin receptor 2A (5HT-2A) were successfully discovered, even though they were not included in the DrugBank database and were thus not in the training set. As expected, the positive drug pairs seemed to be helpful for predicting new targets (e.g. α1 of Motilium, α2 of Xenazine and δ of prantal) by annotation transfer based on the shared target. Interestingly, the newly discovered targets (bold) and those targets not annotated in the DrugBank (underlined) could also be discovered by the new DRS predictions.

As another case study, we tried to find the off-targets of celecoxib (DB00482), which has been known to show unexpected nanomolar inhibition to carbonic anhydrase 2 [24,25], an effect which was not annotated in the DrugBank database. As expected, the known targets of celecoxib appeared in the predicted target list based on positive drug pairs, but carbonic anhydrase 2 could be found only from the newly predicted drug pairs (score 0.826, first rank). In addition, recent studies have shown that celecoxib blocks human cardiac voltage-gated potassium channels (Kv), which accounts for the drug’s known cardiovascular side effects [26,27]. Indeed, the target predictions of celecoxib resulted in a high score for the potassium channels, such as potassium voltage-gated channel subfamily C member 4 (0.505), potassium voltage-gated channel subfamily KQT member 1 (0.451), and potassium voltage-gated channel subfamily E member 1 (0.451). Note that the range of the DRS is from -1 to 1.

### Correlation with another drug similarity score

Campillos et al. calculated the target-sharing probabilities of drugs based on the similarity of side effects and chemical structure [21]. Because both the target-sharing probability and the DRS prioritized drug pairs with common targets, we compared the two methods for each drug group. In the previous study [21], drug pairs with at least 25% probability of sharing a protein target were selected and divided into five groups: the first group (G1) was drug pairs known to share targets (true positives in our study); the second (G2) was drug pairs with similar structures or targets; the third (G3) was drug pairs without known human targets; the fourth (G4) was drug pairs from the same therapeutic category; and the last (G5) was drug pairs predicted only by the side effect similarities.

Pearson’s product-moment correlation coefficient was used to test the significance of the correlation between the two methods. Because the G1 group was drug pairs that shared a target and were included in the training set, the score by our method should obviously be high. On the other hand, all of the drug pairs in other groups were new predictions, so the significant correlations between the two scores seemed to be meaningful. Specifically, the correlation coefficients in G2, G4, and G5 were 0.688 (p-value 1.74e-07), 0.724 (2.85e-05), and 0.396 (2.41e-05), respectively (Additional file 3). Note that the G3 group was not considered because of the insufficient number (eight) of drug-pairs in the group. Accordingly, the two scores are largely correlated to each other even though they use different information.

### Pharmacological effect similarity by drug-drug relationship score

**Figure 4:**
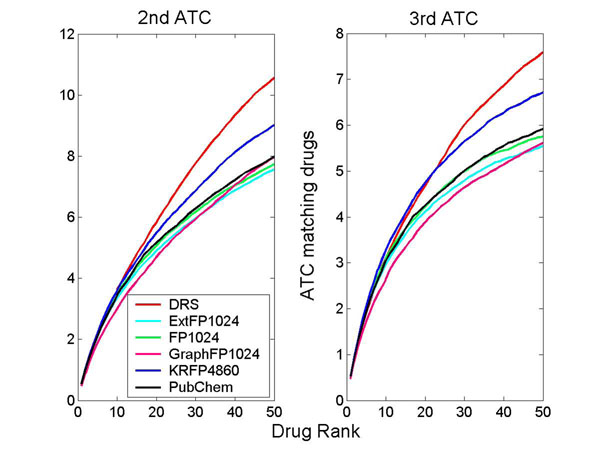
Average numbers of ATC-matching drugs are plotted according to the drug ranks by the DRS. The other drug similarity measures are compared with the DRS. On the left, only exact matches up to 2^nd^ ATC terms are considered, whereas on the right, matches up to 3^rd^ ATC terms are considered.

How much does the DRS represent the actions of drugs? To answer this question, the DRS was used to estimate the similarity of pharmaceutical effects between drugs. For this, the Anatomical Therapeutic Chemical (ATC) system was adapted (http://www.whocc.no/atc/). The ATC system divides drugs into different groups according to the organ or system on which they act, as well as their therapeutic and chemical characteristics. Reflecting the hierarchical structure of the ATC system, the terms of the 2^nd^ and 3^rd^ ATC level were considered to see if the DRS correlated with the pharmacological effect similarity. Specifically, the drug pairs used in the external validation set (i.e. unseen data) were sorted by different drug similarity measures, and the number of drugs with matching ATC was plotted according to that score (Figure 4). We found that the correlation between the DRS and ATC terms was greater than that of drugs with typical structural similarities. The trend did not change when only negative drug pairs (without a shared target) were considered (Additional file 4).

## Conclusions

Chemical similarity has frequently been used to estimate relationships between drugs. For example, in the drug discovery process, the chemical library can be scanned with a query drug to find those compounds which bind to the same target as the query. This drug/target activity view point led us to develop a new target-centric drug-drug relationship score (DRS) under the assumption that drugs that bind with a common target have other common factors. Indeed, the DRS was shown to be closely related to similarities in pharmacological effects.

In our method, to represent drug pairs with their target information, the estimation of drug-drug relationships was restated as a large-scale classification problem that distinguished drug pairs with a common target. In addition, the classification model was improved through data cleaning, iterative under-sampling, and an ensemble approach in combination with a Random Forest classifier. The usefulness of the DRS was demonstrated with internal and external validations, as well as a high domain matching ratio for the new predictions, successful identifications of unknown targets, and a meaningful correlation with another cutting-edge method for studying drug-similarity.

## Methods

### Drug-target interaction data

Drug structure and data on target and drug-target interaction were retrieved from the *DrugBank* database (April 2011) [28]. After erroneous drugs were removed during the descriptor calculation by PaDEL [29], the number of remaining drugs and drug-target interactions were 5,858 and 14,490, respectively. The simple network properties of the relationship are shown in Additional file 5. See the previous work by Yildirim et al. for detailed network properties of the drug-target network [30].

### Drug representation by molecular descriptor

Molecular descriptors (descriptors) are a result of standardized numerical calculations, and logical, mathematical interpretations of chemical information. To characterize drugs, descriptors were calculated using PaDEL software [29]. Specifically, PaDEL descriptors (801), PubChemFP (PubChem fingerprint, 881), EStateFP (E-State fragments, 79), MACCSFP (MACCS keys, 166) and SubFPC (SMART patterns for functional group classification, 307) fingerprints were calculated for each drug. In this procedure, descriptors that generated calculating errors or gave almost the same values for more than 90% of drugs were removed. As a result, 89,354 target-sharing drug pairs were selected as positives, and represented in descriptor space. The drugs were then projected into the largest 162 principal components (PCs), which cumulatively explained 90% of the variance. The purpose of considering the major principal components was to eliminate noise and remove redundant information derived from inter-correlations between descriptors.

### Construction of the drug pair vector

A feature vector representing a drug pair was constructed from the PC-based drug representation (Figure 1). The drug pair vector consisted of an M and an E vector, where the M vector (constructed by averaging PCs between drugs) represents the basal chemical properties and the E vector (obtained by calculating the squared-errors of PCs) represents the chemical property differences. Accordingly, the drug pair vector represented the basal chemical properties and their differences.

### Generation of the drug-drug relationship score from classification model

Another problem of tackling the classification was the proliferation of negative samples as compared to the positive samples, which raised the question of imbalance. When all the samples were used, the number of negative samples was about 200 times larger than the positive samples. Thus, the negatives should be under-sampled, because machine learning techniques usually seek to minimize total prediction errors, so the classification for the imbalanced data tends to be biased towards larger samples.

To minimize the problem, only positive samples were kept, whereas the iterative under-sampling procedure was used to construct multiple negative sample sets. First, the density of structure similarity between drugs was obtained by calculating the PubChem structure similarity for all negative drug pairs. After that, a number of negative drug pairs equivalent to the number of positive drug pairs (89,236) was chosen, based on the sampling probability (inversely proportional to the density of structural similarity). This procedure aimed to select more diverse negative drug pairs, so as not to be biased to specific drug groups. The above procedure was repeated ten times to obtain ten negative sample sets. Then, ten Random Forest classification models were constructed respectively with the positive samples. Finally, the classification scores for the ten classification models were averaged, and the result was regarded as the final drug-drug relationship score. This technique aimed to give a higher score to common-target drug pairs, and ranged from -1 to 1. Note that, to guarantee an “unseen” test set, the score from a single classifier was only used to estimate the classification performance, whereas the average score from the ten classifiers was applied to predict new drug targets.

In the study, Random Forest was used to construct the classification models. Random Forest, developed by Leo Breiman and Adele Cutler, is a collection of tree-based classifiers which constructs trees depending on an independent feature-sampling procedure [31]. Each tree is built by sampling with a replacement, so that about one-third of samples are left out. These OOB (out-of-bag) samples are used to get an unbiased estimate of the classification error. The voting results from an ensemble of decision trees determine the most popular objective class. The Random Forest classifier has been shown to be relatively free from the over-fitting problem as compared to other machine learning methods.

### Validation of classification performance

Two approaches were used to estimate the classification performance. The first of these was internal cross-validation using out-of-bag (OOB) samples from Random Forest classifiers. Random Forest performs a type of cross-validation in parallel with the training step by using out-of-bag (OOB) error estimate. Specifically, the samples that are left out (about one-third of samples) after bootstrapping in the training step become OOB samples. Because these OOB samples have not been used in the tree construction, they can be used to estimate test set errors (OOB error).

In addition, external validation using an independent test set was adopted to estimate the general prediction error of the unseen data. Prior to the training procedure, 50 drugs were randomly selected, and all drug-pairs that included any of those 50 drugs were removed from the training data. After the training procedure, the resulting classifier was tested against the remaining drug pairs. This procedure was used to generate a test set consisting of unseen drug data, and to mimic the virtual screening procedure scanning the most similar drug in the chemical library. The performances of the internal and external cross-validation were shown by a sensitivity-specificity plot. Sensitivity is defined as TP/(TP+FN) and specificity is TN/(TN+FP), where TP is a true positive, FN is a false negative, TN is a true negative, and FP is a false positive.

### Drug structural similarity by various fingerprints

In the present study, 881-bit PubChem fingerprint with the Tanimoto coefficient (ratio of intersection-bits to union-bits) was regarded as a basic measure for chemical structural similarity. In addition, 1024-bit ExtFP (Extends the Fingerprint with additional bits describing ring features), 1024-bit FP (Fingerprint of length 1024 and search depth of 8), 1024-bit GraphFP (specialized version of the Fingerprint which does not take bond orders into account), and 4860-bit KRFP (presence of chemical substructures) calculated from PaDEL software were also used to compare the performance between different fingerprints. To estimate the performance, drug pairs were sorted by the Tanimoto coefficient using different fingerprints to check if the two drugs shared the same target (Figure 2).

### Prediction of potential targets by the drug-drug relationship score

We developed a drug target prediction scheme based on the DRS. The target score for the query drug was obtained by transferring the DRS between the query drug and a drug in the database that binds to the same target. When there were more than two database drugs that bind to the target, the higher DRS (between the query and database drugs) was assigned as the target score. In addition, if the targets had the same score, the one which was more frequently above the predefined score (0.5) came first.

## Competing interests

The authors declare that they have no competing interests.

## Authors’ contributions

KP and DK designed methods, analyzed the data, interpreted the results and wrote the paper.

## Supplementary Material

Additional file 1 Drug structure similarity histogram for true positive drug pairs (correctly predicted positive drug pairs). Click here for file

Additional file 2 Average success rate for the (known) target identification is shown according to the target rank. The target rank is by the target score and the success ratio represent that the score finds the known targets within the corresponding rank (x-axis). Click here for file

Additional file 3 Correlation between the DRS and the drug similarity score from side effect (SE) information. Click here for file

Additional file 4 Average numbers of ATC-matching negative drugs are plotted according to the drug ranks by the DRS. All descriptions are the same to Figure 2. Click here for file

Additional file 5 Simple statistics about drug-target interactions are shown. Click here for file
