# Supplementary figures and images for: Drug-drug relationship based on target information: application to drug target identification
Source: BMC Syst Biol. 2011 Dec 14;5(Suppl 2):S12. doi: 10.1186/1752-0509-5-S2-S12 (PMC3287478; doi:10.1186/1752-0509-5-S2-S12)

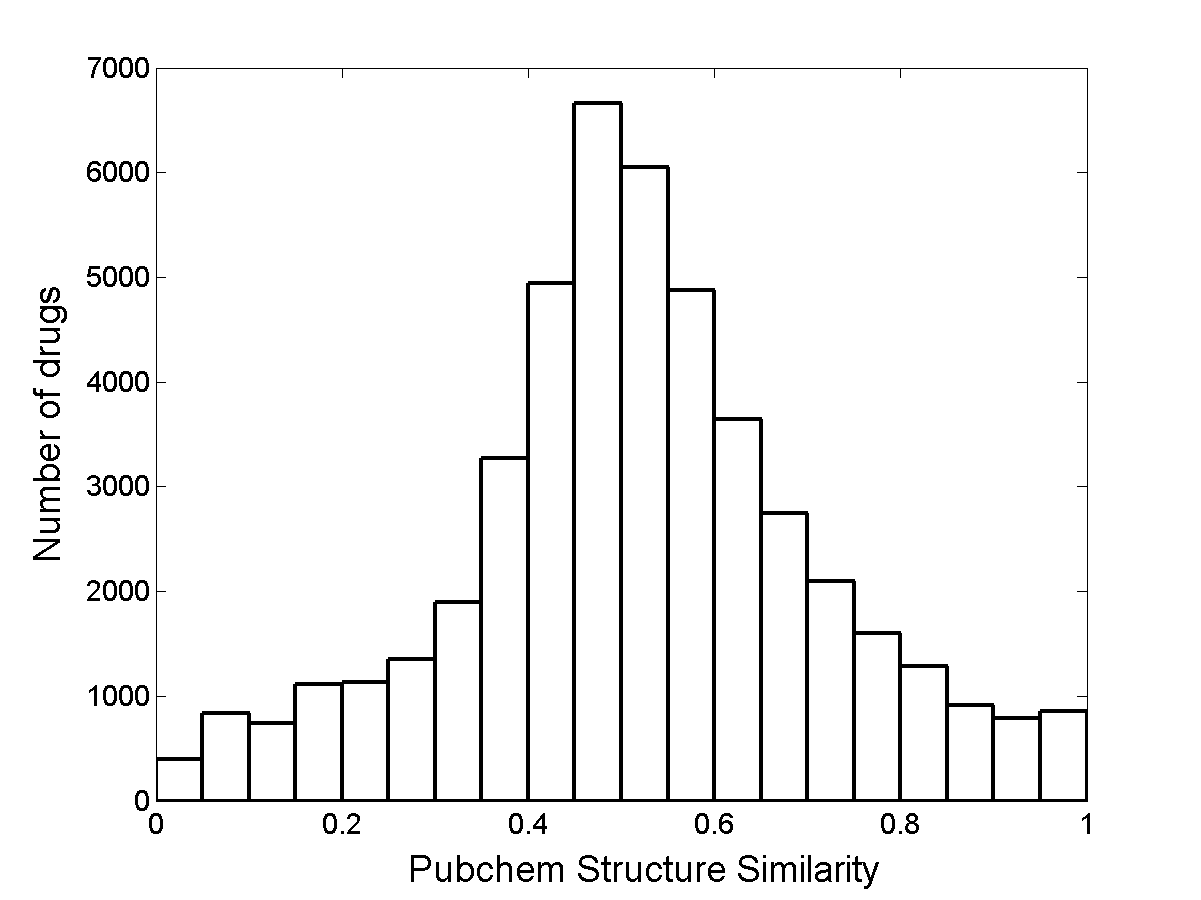

Supplement: Additional file 1 — Drug structure similarity histogram for true positive drug pairs (correctly predicted positive drug pairs). [file 1752-0509-5-S2-S12-S1.tif]

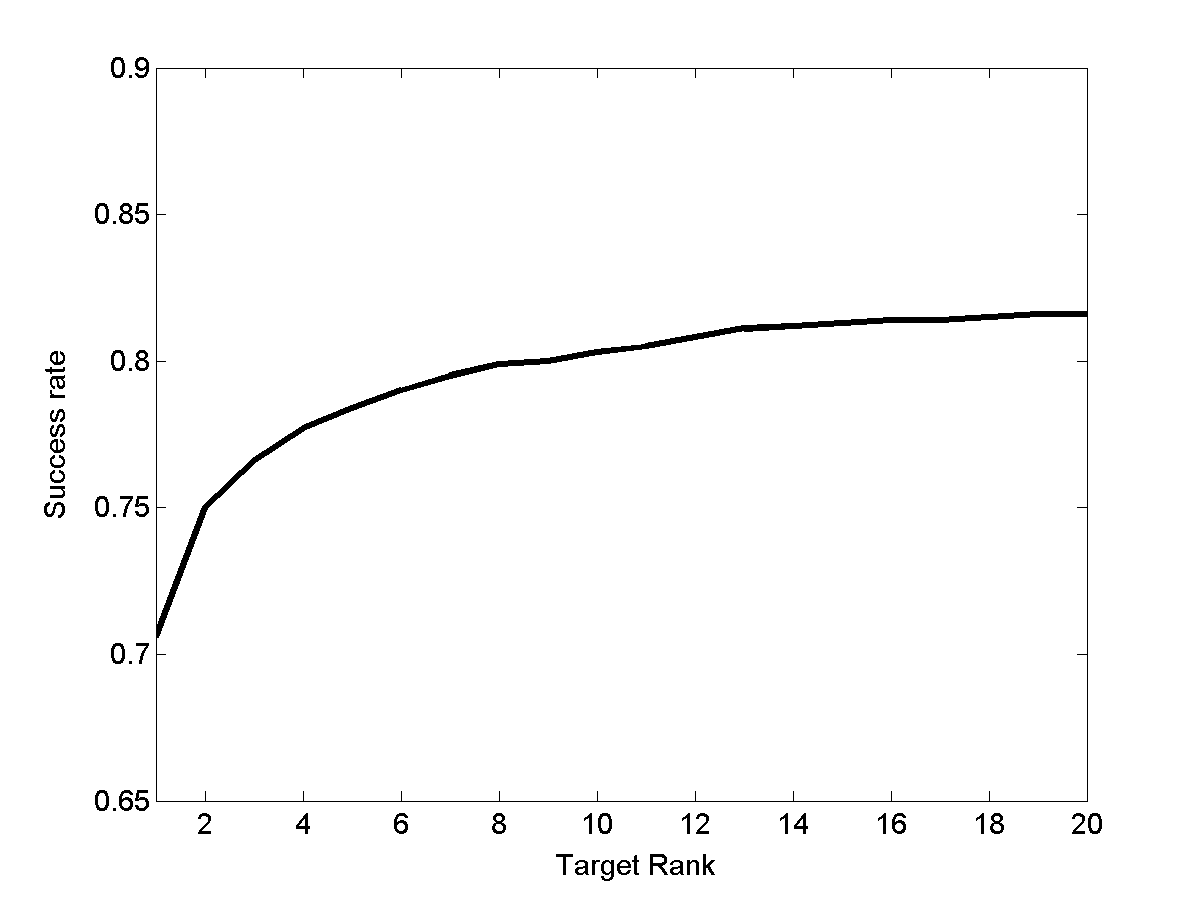

Supplement: Additional file 2 — Average success rate for the (known) target identification is shown according to the target rank. The target rank is by the target score and the success ratio represent that the score finds the known targets within the corresponding rank (x-axis). [file 1752-0509-5-S2-S12-S2.tif]

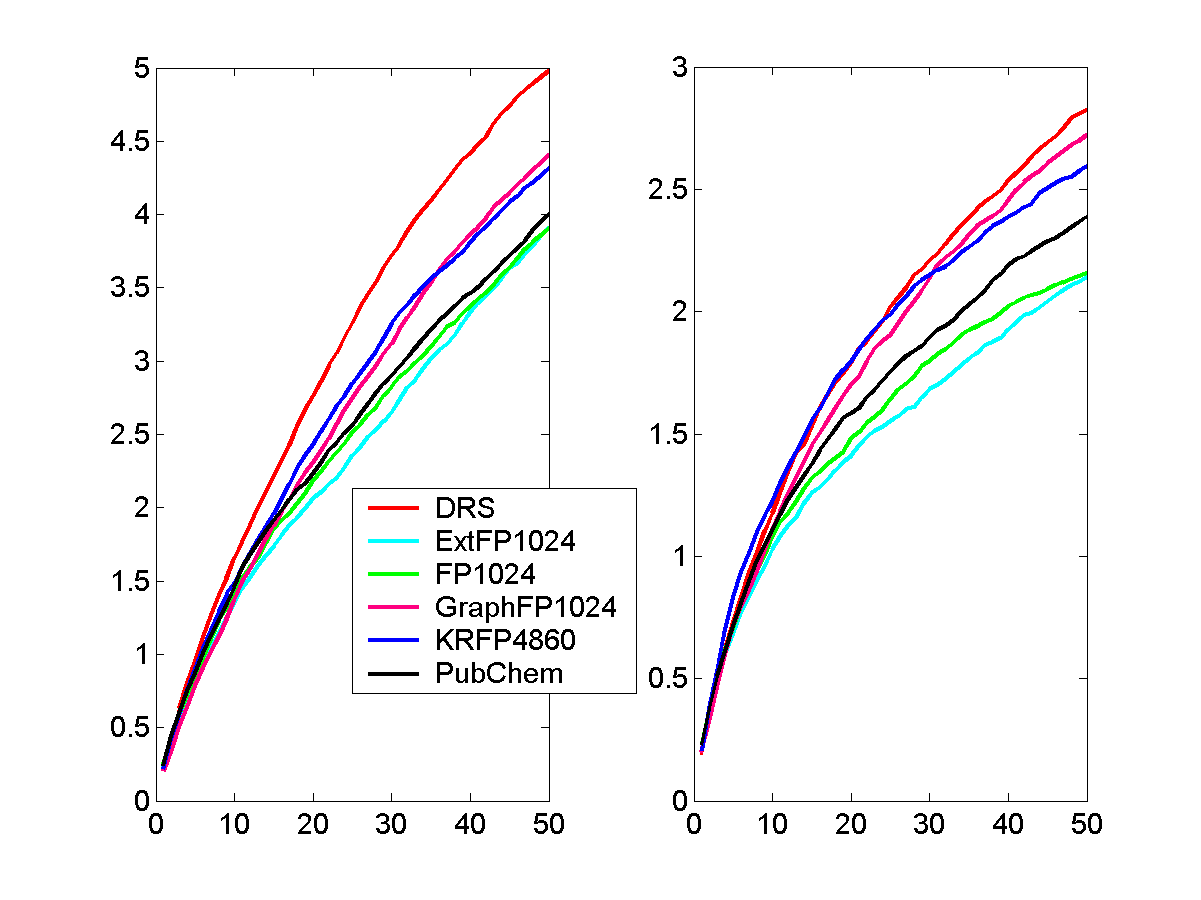

Supplement: Additional file 4 — Average numbers of ATC-matching negative drugs are plotted according to the drug ranks by the DRS. All descriptions are the same to Figure 2. [file 1752-0509-5-S2-S12-S4.tif]
