# Supplementary material for: Drug-drug relationship based on target information: application to drug target identification
Source: BMC Syst Biol. 2011 Dec 14;5(Suppl 2):S12. doi: 10.1186/1752-0509-5-S2-S12 (PMC3287478; doi:10.1186/1752-0509-5-S2-S12)
Supplement: Additional file 3 — Correlation between the DRS and the drug similarity score from side effect (SE) information. [file 1752-0509-5-S2-S12-S3.docx]

**Additional file 3**. Correlation between DRS and the drug similarity score by the side effect (SE) information

| Group | Number | Correlation | p-value | Mean(DRS) | Mean(SE) |
| --- | --- | --- | --- | --- | --- |
| G1 | 184 | 0.352 | 3.206 e-9 | 0.900 | 0.540 |
| G2 | 45 | 0.688 | 5.245 e-6 | 0.703 | 0.516 |
| G3 | 8 | 0.365 | 0.2742 | 0.542 | 0.518 |
| G4 | 26 | 0.724 | 2.176 e-4 | 0.600 | 0.438 |
| G5 | 107 | 0.396 | 6.877 e-5 | 0.509 | 0.375 |
| Total | 370 | 0.514 | < 2.2 e-16 | 0.734 | 0.482 |
