# Supplementary material for: Drug-drug relationship based on target information: application to drug target identification
Source: BMC Syst Biol. 2011 Dec 14;5(Suppl 2):S12. doi: 10.1186/1752-0509-5-S2-S12 (PMC3287478; doi:10.1186/1752-0509-5-S2-S12)
Supplement: Additional file 5 — Simple statistics about drug-target interactions are shown. [file 1752-0509-5-S2-S12-S5.docx]

**Additional file 5** Simple statistics about drug-target interactions

| **Simple data statistics** | **DrugBank Database (2011. 4. 20)** |
| --- | --- |
| # Drugs bound to at least one target | 5,997 |
| # Drugs represented by descriptors | 5,858 |
| # Drug targets | 4,001 |
| # Drug-target interactions | 14,490 |
| # Targets per drug | 3.62 |
| # Drugs per target | 2.41 |
| # Drug pairs | 17,979,006 |
| # Drug pairs bound to the common target | 91,710 |
| # Drug pairs bound to the common target and represented by descriptors | 89,354 |
